# Acid fast bacillus smear, mycobacterial culture and Xpert MTB/RIF assay for the diagnosis of tuberculous peritonitis in patients with and without end stage renal failure

**DOI:** 10.1099/acmi.0.000414

**Published:** 2022-08-11

**Authors:** Richa Misra, Mitra Kar, Samir Mohindra, Amit Gupta

**Affiliations:** ^1^​ Department of Microbiology, Sanjay Gandhi Postgraduate Institute of Medical Sciences, Lucknow, Uttar Pradesh 226014, India; ^2^​ Department of Gastroenterology, Sanjay Gandhi Postgraduate Institute of Medical Sciences, Lucknow, Uttar Pradesh 226014, India; ^3^​ Department of Nephrology, Sanjay Gandhi Postgraduate Institute of Medical Sciences, Lucknow, Uttar Pradesh 226014, India

**Keywords:** culture, diagnosis, tuberculous peritonitis, Xpert MTB/RIF assay

## Abstract

**Introduction.:**

Diagnosis of tuberculous peritonitis (TBP) requires a high index of suspicion.

**Hypothesis /gap statement.:**

Information about the diagnostic features of TBP among patients with end-stage renal failure (ESRF) from India is limited.

**Aim.:**

To assess the utility of the Gene Xpert MTB/RIF assay in the diagnosis of TBP in patients with end-stage renal failure (ESRF), compared with those without ESRF.

**Methodology.:**

This prospective observational single centre cohort study was performed at a tertiary care centre in Northern India. Ascitic fluid and/or whole continuous ambulatory peritoneal dialysis (CAPD) bag with effluent from 300 clinically suspected cases of TBP were included in the study. Diagnosis was based on detection of *Mycobacteria* on smear, Xpert MTB/RIF assay and/or culture. Cell counting was done in a Neubauer chamber. Cell predominance was seen by Giemsa stain. Line probe assay (LPA) for drug susceptibility testing was performed on all positive cultures.

**Results.:**

TBP was diagnosed in 168 cases. Diabetes mellitus was a significant risk factor for developing TBP in patients with ESRF (*P* value<0.01). Lymphocytic predominance was seen in 21 patients without ESRF (*P* value 0.033) while majority of the patients in both groups had neutrophils in their ascitic and peritoneal fluids (138/168; *P* value 0.033). We recovered 15 cases of laboratory diagnosed TBP (11 without ESRF and four with ESRF). Microscopy was positive in two cases while ten isolates were recovered on culture. The Xpert MTB/RIF assay was positive in seven ascitic fluid samples out of which three were rifampicin resistant. All these were patients without renal failure (*P* value 0.010). Eight culture positive samples tested by the line probe assay did not detect any resistance to either rifampicin or isoniazid.

**Conclusion.:**

The GeneXpert MTB/RIF assay has a limited value in the diagnosis of TBP in patients with ESRF.

## Introduction

India is the country with the highest burden of tuberculosis (TB) as well as drug resistant TB. As per the Global Tuberculosis Report 2020, it is the leading cause of death from a single infectious agent worldwide [1].

Tuberculous peritonitis (TBP), although rarer than its pulmonary counterpart, is a serious health concern in regions of the world with high tuberculosis prevalence [1, 2]. TBP is difficult to diagnose clinically, given its insidious onset and nonspecific clinical presentation that often overlaps with many other chronic conditions, such as liver cirrhosis [3]. While medical treatment of the condition is similar to that of pulmonary disease, the generally immune-compromised state of those infected with TBP, along with a lack of highly sensitive and specific testing methods make early diagnosis difficult [4]. The clinical outcome of tuberculous peritonitis therefore depends much on the diagnostic accuracy of this disease entity [5].

The World Health Organization (WHO) in 2010 recommended the Gene Xpert MTB/RIF assay for initial diagnosis of MDR-TB or HIV-associated tuberculosis [6]. In 2014, this recommendation was expanded for use in all patients [7]. To the best of our knowledge, information about the diagnostic features of TBP among patients with end stage renal failure (ESRF) from India is limited. The aim of this study therefore was to assess the diagnostic utility of the Gene Xpert MTB/RIF assay in the diagnosis of TBP in patients with end-stage renal failure (ESRF), compared with those without ESRF. We also aimed to determine the percentage of multi-drug resistant isolates in our patients.

## Methods

### Study design and setting

This prospective observational single centre cohort study between December 2018 to October 2020 was conducted in the Mycobacteriology section of the Department of Microbiology at Sanjay Gandhi Postgraduate Institute of Medical Sciences, a 1200 bed tertiary care referral medical centre in northern India. A junior resident extracted patient data prospectively from the Hospital Information System or files of patients including demographic characteristics, comorbidities, clinical features, and treatments received. Patients with symptoms suggestive of TBP were defined as fever, weight loss, anorexia, the presence of peritoneal effusion, and/or abdominal pain. Definitive TBP cases were defined as those with a peritoneal fluid sample culture that yielded *Mycobacterium tuberculosis* (MTB). Clinically diagnosed TBP cases were defined as those with an exudative ascites, those who showed a clinical improvement after anti-TB treatment and in whom other diagnoses were excluded. The study protocol was approved by the ethics committee of the Institute.

### Clinical specimens and laboratory examination

**Fig. 1.**
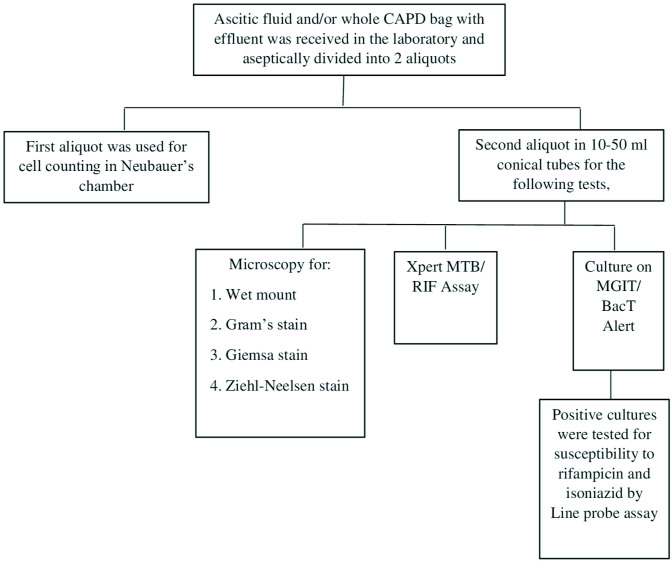
Diagnostic algorithm for samples received in the laboratory (*N*=300).

A total of 300 clinically suspected cases of tuberculous peritonitis (250 ascitic fluid and 50 peritoneal fluid samples), were included in the study. Ascitic fluid samples were collected by paracentesis and received in a syringe or sterile container. For patients on continuous ambulatory peritoneal dialysis (CAPD), the entire bag of effluent was received in the laboratory. The injection port of the bag was first cleaned with 70 % alcohol and fluid withdrawn with a sterile syringe and cannula. Gross examination of the samples was performed prior to processing. Only visibly cloudy fluids were included for cell counting and processing. At least 50 ml of the CAPD fluid and maximum volume of ascitic fluid sample was taken aseptically in two sterile 50 ml conical centrifuge tubes. The tubes were centrifuged at 3 000 r.p.m. for 30 min or 5 000 r.p.m. for 20 min. The supernatant was discarded and the sediment was further processed for culture. Fig. 1 shows the diagnostic algorithm for samples received in the laboratory. One millilitre of the uncentrifuged sample was used for cell counting. Wet mount, Gram’s, Giemsa, and Ziehl–Neelsen stained smears were prepared for microscopic examination. One aliquot of the sample was used to perform the Xpert MTB/RIF assay as per manufacturer’s protocol (Gene Xpert Instrument Systems, Cepheid). Culture for *Mycobacteria* was performed by the *N*-acetyl-Lcysteine-sodium citrate-NaOH (NALC-NaOH) method. Samples were decanted following centrifugation, and sediments were re-suspended in 3 ml of phosphate buffer solution. Processed samples were used to inoculate either Lowenstein–Jensen (LJ) solid medium or BacT/Alert culture. Line probe assay *version2* (LPA*v2*) was used for testing susceptibility to isoniazid and rifampicin from positive cultures.

### Statistical analysis

All continuous variables were expressed as median and range. Comparisons of categorical variables between patients with ESRF and without ESRF were conducted using the Chi-square test. All calculations in this study were conducted with IBM SPSS Statistics version 20.0 software (IBM Corp., Armonk, NY, USA). A *P*-value of less than 0.05 was considered as significant.

## Results

**Fig. 2.**
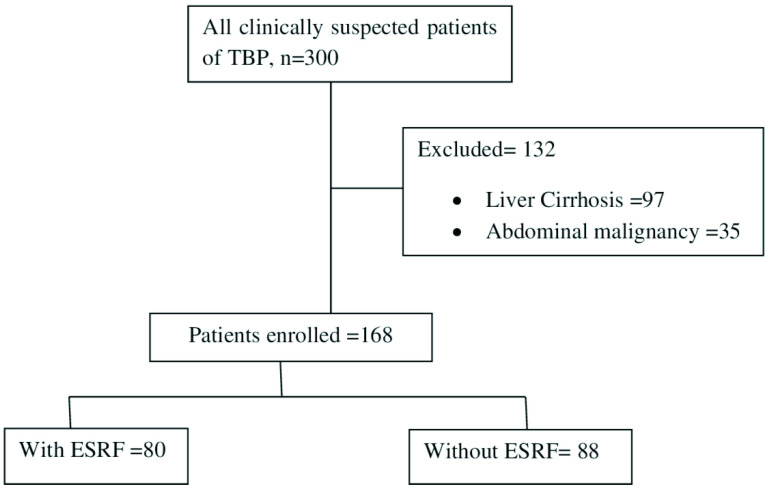
Flow chart of patient enrolment.

During the study period, 300 patients were included in the study. Out of these, 132 patients were excluded as shown in Fig. 2. These included 97 patients of liver cirrhosis and 35 with abdominal malignancy. TBP was diagnosed in 168 cases. The demographic characteristics and clinical presentation of these patients is shown in Table 1. Eighty patients had ESRF, 46 of whom underwent CAPD. The age difference between both the groups was statistically significant (*P* value <0.001). Diabetes mellitus was a significant risk factor for developing TBP in patients with ESRF (*P* value <0.01). Abdominal distension (83 %) followed by abdominal pain (76 %) was the predominant clinical presentation in majority of the patients. Patients with ESRF had a more acute presentation than did patients without ESRF (*P* value <0.005). Fever was equally distributed among both the groups. There was statistically no significant difference between both groups for clinical presentations like anorexia, weight loss, vomiting, constipation, diarrhoea or night sweats. Co-existing pulmonary TB was diagnosed in 26 cases but the numbers were distributed equally among both groups. The laboratory results of tuberculosis (TB) peritonitis among patients with and without end-stage renal failure are summarized in Table 2. A total of 122 ascitic fluid (AF) and 46 peritoneal dialysis fluid (PD) samples were included in the study. Majority of the patients in both groups presented with cloudy fluids (*P* value 0.009). The fluids were blood stained in 14 samples and this difference was statistically significant among both groups (*P* value 0.040). The median WBC count was only slightly higher in patients without ESRF. Lymphocytic predominance was seen in 21 patients without ESRF (*P* value 0.033) while majority of the patients in both groups had neutrophils in their ascitic and peritoneal fluids (138/168; *P* value 0.033). We recovered 15 cases of laboratory diagnosed TBP (11 without ESRF and four with ESRF). Microscopy was positive in two cases while ten isolates were recovered on culture (eight AF and two PD fluid samples). The Xpert MTB/RIF assay was positive in seven ascitic fluid samples out of which three were rifampicin resistant. One case was rifampicin indeterminate. All these were patients without renal failure (*P* value 0.010). Eight culture positive samples tested by the Line probe assay did not detect any resistance to either rifampicin or isoniazid. A summary of the performance data for GeneXpert MTB/RIF assay, MTBC culture and smear results is shown in Table 3. The GeneXpert assay had a 70% agreement for culture positive specimens for the detection of *M. tuberculosis *.

**Table 1.** Demographic characteristics of patients and risk factors for clinically suspected tuberculous peritonitis (TBP) with and without end-stage renal disease (ESRD), (*n*=168)

	With ESRD (*n*=80)	Without ESRD (*n*=88)	*P*-value
Age, median years (range)	49.50 (7–84)	28.50 (1–70)	<0.001*
Gender, male :female	47 : 33	49 : 39	0.688
Diabetes mellitus	44 (55.0 %)	15 (17.04 %)	<0.01*
Clinical presentation			
Abdominal distension (*n*=138)	67 (83.75 %)	71 (80.68 %)	0.604
Abdominal pain (*n*=110)	61 (76.25 %)	49 (55.68 %)	<0.005*
Fever (*n*=106)	54 (67.5 %)	52 (59.09 %)	0.259
Anorexia (*n*=69)	29 (36.25 %)	40 (45.45 %)	0.226
Weight loss (*n*=48)	23 (28.75 %)	25 (28.40 %)	0.961
Vomiting (*n*=28)	11 (13.75 %)	17 (19.31 %)	0.333
Constipation (*n*=19)	7 (8.75 %)	12 (13.63 %)	0.318
Diarrhoea (*n*=17)	6 (7.50 %)	11 (12.5 %)	0.283
Night sweats (*n*=15)	7 (8.75 %)	8 (9.09 %)	0.938
Co- existing TB			
Extrapulmonary TB	14 (17.50 %)	19 (21.59 %)	0.379
Pulmonary TB	13 (16.25 %)	13 (14.77 %)	0.791

**Table 2.** Laboratory findings of tuberculous peritonitis (TBP) among patients with and without end-stage renal disease (ESRD) (*n*=168)

Test result	With ESRD (*n*=80)	Without ESRD (*n*=88)	*P*-value
Ascitic and peritoneal fluid specimen analysis			
Gross examination			
Cloudy	78 (97.50 %)	76 (86.36 %)	0.009
Blood stained	2 (2.50 %)	12 (13.63 %)	0.040*
Total WBC count, median cells mm^−3^ (range)	196 (102–2765)	212 (114–3490)	0.054*
Lymphocytic predominant	9 (11.25 %)	21 (23.86 %)	0.033*
Neutrophil predominant	71 (88.75 %)	67 (76.14 %)	0.033*
Positive AFB smear results	0 (0.00 %)	2 (2.27 %)	0.175
Positive AFB culture results	4 (5.00 %)	6 (6.81 %)	0.619
Positive results for Gene Xpert MTB/RIF Assay	0 (0.00 %)	7 (7.95 %)	0.010*

**Table 3.** Performance data for microscopy, Gene Xpert MTB/RIF assay and MTBC culture (*n*=168)

Smear results (*n*=168)	No. of assay results				
	MTB culture +, GeneXpert +	MTB culture +, GeneXpert −	MTB culture −, GeneXpert+	MTB culture −, GeneXpert -	Total
Positive	0	1	1	0	2
Negative	2	7	4	153	166
Total	2	8	5	153	168

## Discussion

This is the first study from India to assess the diagnostic utility of the Gene Xpert MTB/RIF assay in the diagnosis of tuberculous peritonitis in such a large series of patients with and without end-stage renal failure. The rate of laboratory confirmed TBP in our cohort was 8.9 % among 168 cases of clinically suspected TB peritonitis which is alarmingly high. Though TBP can occur at any age, it is predominantly a disease affecting young adults in the third and fourth decades of life [3]. The overall median age of our patients was 37 years while in the ESRF group it was 49.5 years. This is in contrast to the study by Chau *et al.* where the median age of patients was 62 years [8]. The Indian Chronic Kidney Disease (CKD) registry has also reported the mean age of patients as 50.1±14.6 years with diabetic nephropathy as the commonest cause of ESRF.

Diabetes, which can lead to the need for CAPD, is also a known risk factor for tuberculosis. In a study from Taiwan, the incidence of TBP in diabetics was as high as 26.7 %, as opposed to 6.7 % in non-diabetics [9]. We too found a significant association of diabetes as a risk factor for acquiring TBP in patients with ESRF (*P* value<0.01).

The clinical presentation of TB peritonitis can be very nonspecific [10]. The classic triad of abdominal pain, abdominal distension (ascites), and fever occurred in only 31.5 % patients of our study cohort. Several published series have reported the two commonest symptoms to be abdominal pain (31–94 %) and fever (45–100 %) [3, 10, 11]. Patients in our study with ESRF tended to present more commonly with abdominal pain than patients without renal failure. The acute presentation along with the presence of neutrophilia may mimic acute bacterial peritonitis [8]. In fact, concurrent bacterial peritonitis was present in 21 patients in our study and this could mask the clinical features of TB peritonitis.

The WBC cell counts in TBP can range widely from 100 cells mm^−3^ to as high as 5000 cells mm^−3^. In a case report and review by Talwani and Horvath, 78 % of patients had PMN-predominant pleocytosis on examination of the peritoneal fluid [12]. Generally, counts range from 500 to 1500 cells per mm^3^ [13, 14]. Chow *et al.* in a study on cirrhotic patients with tuberculous peritonitis observed polymorphonuclear leucocyte–predominant ascites [5]. We observed predominance of neutrophils in both groups and higher counts of lymphocytes in 21 patients without ESRD and this difference was statistically significant. Various studies have reported predominance of lymphocytes in peritoneal fluid except in patients with renal failure [8]. Such patients may in fact be misdiagnosed as spontaneous bacterial peritonitis or ‘culture negative neutrocytic ascites’.

A significant proportion of patients with TBP have coexistent pulmonary tuberculosis as well [10]. Latent foci of infection established in the peritoneum get reactivated via haematogenous spread to the mesenteric lymph nodes from a current or previous pulmonary infection. However, data for association of TBP with pulmonary TB is significantly different between the western and eastern hemispheres. A study from the United States by Sieloff *et al.* investigated hospitalizations for tuberculous peritonitis from 2002 to 2014 and found that only 6.37 % of TBP admissions were associated with pulmonary TB [15]. On the contrary, Manohar *et al.* in their study on 145 patients with tuberculous peritonitis reported active pulmonary disease in 17.9% (*n*=26) patients [10]. Though 26 patients in our study had pulmonary TB, we could not find an association of its presence with the risk of acquiring TBP in patients with or without renal failure. Patients with ESRD are a high-risk group for getting TBP. They have an impaired cell-mediated immune response which can predispose to infection though the exact portal of entry of *M. tuberculosis* into the peritoneum remains unclear [8]. Direct contamination via peritoneal dialysis can be one of the mechanisms of infection. Lui *et al.* conducted a retrospective study on the prevalence and pattern of tuberculosis in patients undergoing continuous ambulatory peritoneal dialysis (CAPD). In their series, 14 of 790 patients on CAPD were diagnosed with peritoneal TB between 1994 and 2000 [16]. CAPD has a strong association with developing TBP in the United States and similar studies have been reported from Taiwan and Turkey as well [9, 17]. We diagnosed two cases of CAPD peritonitis by culture in our cohort and both required removal of catheter. In two case histories described by Edwards *et al.* diagnosis was established by the Xpert MTB/RIF Assay and treatment was successful without hemodialysis. PD was continued with a successful outcome [18]. A review of literature has revealed that removal of the PD catheter in TB peritonitis cases is controversial. While some authors propose catheter removal and re-insertion after 6 weeks of anti-tuberculous treatment, there are other reports of successful treatment without catheter removal [6].

The diagnosis of TBP requires a high index of clinical suspicion due to the paucibacillary nature of the disease [13, 14]. The current gold standard is laparoscopy and peritoneal biopsy with microbiological or pathological confirmation by microscopy and culture for acid-fast bacilli [13].

In addition, in TB endemic areas the clinicians are often faced with the difficulty of differentiating tuberculosis and Crohn’s disease. The absence of a good sensitive and specific confirmatory test necessitates a therapeutic trial of ATT. This is a very common practice in India. However, at the end of an ATT trial, if a patient has not responded and mucosal inflammatory changes are still seen on colonoscopy, the suspicion of MDR-TB arises. In a recent study from our centre, we assessed the burden of TB in a large cohort of consecutive patients over a period of 2 years at our centre and the rate of rifampicin resistance in pulmonary samples was 23.5 % while in extrapulmonary cases, it was 17.4 % [19].

Ziehl–Neelsen (ZN) staining of ascitic fluid is positive in only about 3 % of cases with proven TBP [18]. The yield from culture even with a combination of both liquid and solid media is less than 50 % and it requires a minimum of 2–8 weeks [4]. The delay in diagnosis and the associated high mortality rate of 50–60% underscores the urgent need for new laboratory methods for the diagnosis of TBP, especially in countries with a high burden of TB as well as MDR-TB [5]. The Xpert MTB/RIF assay is a multiplex hemi-nested real-time PCR-based technique to detect presence of *M. tuberculosis* within 2 h [6, 7]. In our study the assay was positive in seven out of 168 cases of clinically suspected cases of TBP, out of which only two grew on culture. It therefore had only a 25% agreement for culture positive, smear-positive specimens. This could be due to the fact that positive results yielded by Xpert assay are based on the presence of bacterial DNA rather than live tubercle bacilli required by mycobacterial culture, which may result in false-negative cultures. Similar results have been reported by other authors. Liu *et al.* in a study on 191 cases presenting with symptoms suggestive of TBP reported sensitivities of MGIT culture and Xpert as 17.2 and 18.3%, respectively [20]. In a report by Bera *et al.* on the use of GeneXpert in peritoneal TB, the test was positive in only four out of 21 patients [21]. Similarly, only three out of 37 patients had a positive Xpert MTB/Rif test in a study on intestinal TB and its differentiation from Crohn’s disease, suggesting that it was of limited use in diagnosing peritoneal tuberculosis [22]. In another study, the diagnostic yield of Xpert MTB/RIF assay on ascitic fluid samples was lower than MGIT-960 culture (17.9% versus 25.5%) [23]. However, two cases of tuberculous peritonitis have been reported in patients on PD where a prompt diagnosis was made employing the Xpert MTB/RIF assay.

We also found that the Gene Xpert assay had lower sensitivity than culture for the diagnosis of TBP in patients with ESRF. None of the patients with ESRD had a positive Xpert result and this difference was statistically significant. In a recent meta-analysis [24] which included 25 observational studies, the authors concluded that the Xpert MTB/RIF assay has modest sensitivity for diagnosis of peritoneal and intestinal tuberculosis but has a good specificity. Another study by Mor *et al.* also suggested that among various NAATs, such as *IS6110* nested PCR, M-PCR targeting *mpt64 +IS6110* and *mpt64 +IS6110+pstS1*, the Xpert MTB/RIF assay is an excellent rule-in test but is not a good rule-out test [25].

We also documented a high rate of drug resistance in our study. Out of seven samples positive by the Xpert assay, three were rifampicin resistant. High rates of drug resistance have been reported by us from our centre and TB is one of the top ten causes of death as per the Global Tuberculosis Report published by WHO in 2020 [1, 19]. A study by Law *et al.* on the emergence of drug resistance in tuberculosis patients cared for by the Indian health-care system estimated that by 2032, 85% of multidrug-resistant tuberculosis will be primary compared with only 15 % in 2012 [26]. They conclude that effective treatment of drug-susceptible tuberculosis will not curb the spread of the disease as it has transformed to a drug-resistant epidemic in India.

This epidemiological shift has profound resource implications since the cost of treatment of multidrug-resistant tuberculosis can exceed that of first-line tuberculosis therapy by a factor of ten or more. The situation in India is further aggravated by a severe lack of microbiology laboratories and a complex health-care system consisting of government and private allopathic providers, pharmacists, ayurvedic and homoeopathic practitioners who freely provide over the counter antibiotics.

## Conclusions

The diagnosis of TB peritonitis requires a high index of suspicion. Our study demonstrates the difficulty in establishing a diagnosis of TB peritonitis in patients with ESRF and limited value of the GeneXpert MTB/RIF assay. In patients with ESRF who have TB peritonitis, neutrophil-predominant peritoneal fluid is common.

The most obvious limitation of our study was the lack of definitive diagnosis by peritoneal biopsy. However, our observations are prospective and have significant implications for a high TB burden country like India.
